# *CYLD* mutation characterizes a subset of HPV-positive head and neck squamous cell carcinomas with distinctive genomics and frequent cylindroma-like histologic features

**DOI:** 10.1038/s41379-020-00672-y

**Published:** 2020-09-05

**Authors:** Erik A. Williams, Meagan Montesion, Brian M. Alexander, Shakti H. Ramkissoon, Julia A. Elvin, Jeffrey S. Ross, Kevin Jon Williams, Krzysztof Glomski, Jacob R. Bledsoe, Julie Y. Tse, Mark C. Mochel

**Affiliations:** 1 Foundation Medicine, Inc., 150 Second Street, Cambridge, MA 02141 USA; 2 Wake Forest Comprehensive Cancer Center and Department of Pathology, Wake Forest School of Medicine, Winston-Salem, NC 27157 USA; 3 Department of Pathology, State University of New York Upstate Medical University, 766 Irving Avenue, Syracuse, NY 13210 USA; 4 Department of Physiology, Department of Medicine, Lewis Katz School of Medicine at Temple University, Philadelphia, PA 19140 USA; 5 Hartford Pathology Associates, 80 Seymour Street, Hartford, CT 06102 USA; 6 Department of Pathology, University of Massachusetts Medical School, One Innovation Drive, Worcester, MA 01605 USA; 7 Departments of Pathology and Dermatology, Virginia Commonwealth University School of Medicine, 1200 East Marshall Street, Richmond, VA 23298 USA

**Keywords:** Oral cancer, Tumour virus infections

## Abstract

Mutations in the tumor suppressor *CYLD*, known to be causative of cylindromas, were recently described in a subset of high-risk (hr) HPV-positive head and neck squamous cell carcinomas (HNSCC). Pathologic and genetic characterization of these *CYLD*-mutant carcinomas, however, remains limited. Here, we investigated whether *CYLD* mutations characterize a histopathologically and genomically distinct subset of hrHPV-positive HNSCC. Comprehensive genomic profiling via hybrid capture-based DNA sequencing was performed on 703 consecutive head and neck carcinomas with hrHPV sequences, identifying 148 unique cases (21%) harboring *CYLD* mutations. Clinical data, pathology reports, and histopathology were reviewed. *CYLD* mutations included homozygous deletions (*n* = 61/148; 41%), truncations (*n* = 52; 35%), missense (*n* = 26; 18%) and splice-site (*n* = 9; 6%) mutations, and in-frame deletion (*n* = 1; 1%). Among hrHPV-positive HNSCC, the *CYLD*-mutant cohort showed substantially lower tumor mutational burden than *CYLD*-wildtype cases (*n* = 555) (median 2.6 vs. 4.4 mut/Mb, *p* < 0.00001) and less frequent alterations in *PIK3CA* (11% vs. 34%, *p* < 0.0001), *KMT2D* (1% vs. 16%, *p* < 0.0001), and *FBXW7* (3% vs. 11%, *p* = 0.0018). Male predominance (94% vs. 87%), median age (58 vs. 60 years), and detection of HPV16 (95% vs. 89%) were similar. On available histopathology, 70% of *CYLD*-mutant HNSCC (98/141 cases) contained hyalinized material, consistent with basement membrane inclusions, within crowded aggregates of tumor cells. Only 7% of *CYLD*-wildtype cases demonstrated this distinctive pattern (*p* < 0.0001). Histopathologic patterns of *CYLD*-mutant HNSCC lacking basement membrane inclusions included nonkeratinizing (*n* = 22, 16%), predominantly nonkeratinizing (nonkeratinizing SCC with focal maturation; *n* = 10, 7%), and keratinizing (*n* = 11, 8%) patterns. The latter two groups showed significantly higher frequency of *PTEN* alterations compared with other *CYLD-*mutant cases (38% [8/21] vs. 7% [8/120], *p* = 0.0004). Within our cohort of hrHPV-positive HNSCCs, *CYLD* mutations were frequent (21%) and demonstrated distinctive clinical, histopathologic, and genomic features that may inform future study of prognosis and treatment.

## Introduction

The most recent World Health Organization classification of head and neck tumors divides squamous cell carcinoma (SCC) of the oropharynx into high-risk (hr) HPV-positive and hrHPV-negative tumors [1]. Among hrHPV-positive head and neck SCC (HNSCC), currently recognized morphologic variants include papillary, adenosquamous, lymphoepithelioma-like, and basaloid subtypes [1]. In addition to carcinomas of the oropharynx, hrHPV-positive SCC also occurs at a lower frequency in other head and neck sites including the sinonasal tract, oral cavity, and hypopharynx and larynx [2].

Nonkeratinizing histomorphology in SCC of the head and neck strongly correlates with HPV infection [3]. The basaloid subtype of oropharyngeal SCC, originally described by Wain et al. [4], contains a basaloid component of crowded cells with scant cytoplasm, often with thickened basement membranes and inclusions of basement membrane material, associated with foci of keratinization [4–6]. Some authors emphasize the “jigsaw-puzzle” pattern of closely apposed tumor lobules, cystic spaces with myxoid material, deposition of basement membrane material, and abrupt keratinization to delineate the basaloid variant of HNSCC from nonkeratinizing HPV-positive SCC, which often displays “basaloid” cytomorphology (i.e., tumor cells with scant cytoplasm and hyperchromatic nuclei) [7, 8]. Basaloid variant SCC with confirmed HPV positivity has shown a better prognosis than HPV-negative basaloid variant SCC [6, 9]. As such, some authorities emphasize HPV testing in SCCs with histologic features of the basaloid variant, to exclude the aggressive HPV-negative basaloid variant [10].

Molecular genetic profiles of HNSCC differ by HPV status [11, 12]. The genes most commonly altered in HPV-negative HNSCC include *TP53*, *CDKN2A*, and *CCDN1*. In contrast, HPV-positive HNSCC demonstrates a low rate of *TP53* and *CDKN2A* mutations, while *PIK3CA* mutations*, TRAF3* loss, and *E2F1* amplifications are relatively common [11].

Mutations of *CYLD*, which encodes an enzyme regulator of NF-κB, have been previously characterized as causative of syndromic and sporadic cylindroma [13, 14] and were recently described in HNSCC [15]. A recent study demonstrated that HPV-positive HNSCC with mutations of either *CYLD* or *TRAF3*, which has similar function to *CYLD*, demonstrated increased NF-κB activity, harbored HPV DNA in episomal form rather than integrated genomic form, and correlated with improved patient survival [15]. Histopathologic features and comprehensive genomic characteristics were not reported for this distinctive molecular subtype of HNSCC.

Given recent work by our group that correlated *CYLD* mutations in HPV-positive basaloid carcinomas of the anus with cylindroma-like histologic features, low tumor mutational burden (TMB), and low frequency of *PIK3CA* driver mutations [16], we hypothesized that *CYLD*-mutant, HPV-positive HNSCCs may have similarly distinctive histologic and genomic features. In the current study, we retrospectively assessed HPV-positive head and neck carcinoma samples analyzed by comprehensive genomic profiling (CGP) for *CYLD* mutations. This analysis may enhance tumor categorization and inform further correlations with prognosis and potential therapeutic approaches to *CYLD*-mutant tumors, such as recently investigated targeted inhibitors for cylindromatosis [17, 18].

## Materials and methods

### Cohort and genomic analyses

Approval for this study, including a waiver of informed consent and Health Insurance Portability and Accountability Act waiver of individual authorization, was obtained from the Western Institutional Review Board (Protocol No. 20152817). In the course of clinical care, physicians at outside institutions sent tumor samples for CGP by our Clinical Laboratory Improvement Amendments-certified, College of American Pathologists-accredited laboratory (Foundation Medicine, Inc., Cambridge, MA, USA). Here, we searched our database of cases with CGP to identify all HPV-positive head and neck carcinomas with *CYLD* mutation versus *CYLD-*wildtype tumors. HPV status of each case was determined via the sequencing platform, as detailed below. We excluded adenocarcinomas and tumors of the salivary glands. In brief, board-certified pathologists confirmed pathologic diagnoses and tissue adequacy for all cases on routine hematoxylin and eosin (H&E)-stained slides before DNA extraction. Macrodissection of sections was performed to achieve an estimated percent tumor nuclei (%TN) above 20% in each case, where %TN is defined as 100 times the number of tumor cells divided by the total number of nucleated cells. For genomic assessment, ≥60 ng of DNA was extracted from 40-μm sections cut from formalin-fixed, paraffin-embedded tissue blocks containing tumor. Adaptor ligation hybrid capture, utilizing the Foundation One T7 baitset [19], was performed on the specimens. Supplementary Table 1 lists all sequenced genes in the T7 baitset. The Illumina HiSeq 4000 System was used to perform sequencing of captured libraries to a mean exon coverage depth of targeted regions of >500X. Sequences were analyzed for genomic alterations, including short variant alterations (base substitutions, insertions, and deletions), copy number alterations (focal amplifications and homozygous deletions), and select gene fusions or rearrangements [19–21]. For maximum sensitivity and specificity of mutation detection in impure clinical specimens, sequencing was previously optimized and validated to detect base substitutions at a ≥5% mutant allele frequency, indels with a ≥10% mutant allele frequency with ≥99% accuracy, and fusions occurring within baited introns/exons with >99% sensitivity [19]. TMB, quantified as mutations per Mb, was assessed on 1.1 Mb of sequenced DNA [21]. Up to 114 loci were assessed for determination of microsatellite instability (MSI) [22]. De novo assembly of nonhuman sequencing reads and comparison utilizing the basic local alignment search tool for nucleotides (BLASTn) against viral sequences in the comprehensive NCBI RefSeq database was used to detect HPV DNA sequences. HPV positivity was designated for cases with contigs ≥80 nucleotides in length with ≥97% sequence identity to the Refseq sequence identified by BLASTn. Evaluated HPV types included HPV6, 11, 16, 18, 26, 31, 33, 35, 39, 40, 42, 43, 44, 45, 51, 52, 53, 54, 55, 56, 58, 59, 61, 62, 64, 66, 67, 68, 69, 70, 71, 72, 73, 81, 82, 83, 84, CP6108, and IS39. HPV16, 18, 33, and 58 were designated hr, and HPV6 was designated low-risk, as previously described [23].

### Mutational signatures

The presence and quality of mutational signatures was evaluated for all samples with at least 20 nondriver missense mutations. Signatures were assigned through analysis of the trinucleotide context with profiling of the Sanger COSMIC cancer mutational signatures [24]. A positive signature required at least 40% of identified genomic alterations to fit to a known mutational process: APOBEC overexpression (C > T and C > G base substitutions), exposure to UV light (C > T and CC > TT base substitutions at dypirimidine sites), hypofunction of the BRCA tumor suppressor (>3 base pair insertions or deletions with overlapping microhomology at breakpoint junctions), and defects in mismatch repair (<3 base pair insertions and deletions at mono/polynucleotide repeats) [24].

### Germline prediction

Computational prediction of whether a particular *CYLD* mutation was germline was performed using a previously validated somatic-germline-zygosity algorithm [20]. This technique aligns sequencing reads and mutant allele frequencies for detected mutations and compares these to the expected values generated by the copy number model [19]. Based on this comparison, the algorithm calculates a prediction of whether the variant was germline, somatic, or ambiguous [20].

### Clinicopathologic analyses of head and neck carcinomas with CYLD mutations

Outside institutional pathology reports that accompanied each specimen sent to Foundation Medicine for CGP were reviewed to collect clinicopathologic data, including patient age, gender, tumor site, and AJCC stage (8th edition) [25].

H&E-stained sections from each available *CYLD*-mutant hrHPV-positive carcinoma were assessed retrospectively by three pathologists (EAW, JYT, MCM) for histologic features, including growth pattern, deposition of basement membrane material, squamous differentiation, necrosis, and cytomorphology. The description of “basaloid” cytomorphology was applied to tumors composed of cells with scant cytoplasm and hyperchromatic nuclei. The designation of carcinomas with “cylindroma-like histopathologic features” was assigned principally by the presence of inclusions of hyalinized material, consistent with basement membrane inclusions reminiscent of those seen in cylindroma, within aggregates of carcinoma cells; however, additional histologic features, described in the “Results,” were also common in this subgroup. The identification of keratinization was based on the histologic presence of squamous pearls or aggregates of cells with glassy, eosinophilic cytoplasm. SCCs lacking basement membrane inclusions were classified into one of three categories: “non-keratinizing” for SCCs lacking keratinization; “predominantly non-keratinizing” for SCCs in which keratinization was present but limited to small foci representing <10% of the examined tumor area; and “keratinizing” for SCCs with keratinization in ≥10% tumor area. Histopathology of *CYLD*-wildtype hrHPV-positive carcinomas was reviewed for comparison. Histopathologic assessment of all cases was limited to the sequenced tissue, representing one block of tumor per case. Reviewers were aware of the *CYLD* status when evaluating the histologic features. Although immunohistochemical slides were not available for retrospective review, we collected immunohistochemical findings from pathology reports.

Given the size of the cohort, Fisher’s exact test was used for analyses of categorical data. For comparison of continuous parameters (age and TMB) between two groups, the non-parametric Mann–Whitney *U* test was used. A two‐tailed *p* value of <0.05 was considered statistically significant. For multiple simultaneous comparisons, the Bonferroni correction was applied.

## Results

### Clinicopathologic features of *CYLD*-mutant hrHPV-positive head and neck carcinoma

In our database, we identified 703 distinct hrHPV-positive head and neck carcinoma specimens, of which head and neck adenocarcinoma and cases of salivary gland origin were excluded as no HPV-positive *CYLD*-mutant cases were identified in these groups. In total, 148 distinct cases (21%) featured *CYLD* mutations. Compared with *CYLD*-wildtype head and neck carcinomas (*n* = 555), *CYLD*-mutant cases showed similar male predominance as *CYLD*-wildtype hrHPV-positive head and neck carcinoma (“*CYLD*-wildtype”; 94% [139/148] vs. 87% [484/555], respectively, not significant with corrected *p* value; Table 1), as well as median age (58 vs. 60 years, NS; Table 1). The cohort of 148 *CYLD*-mutant carcinomas encompassed 40 primary HNSCC, 1 primary-site recurrence, and 107 metastatic samples. The metastatic samples were from the liver (*n* = 35/107; 33%), lung (*n* = 30; 28%), regional lymph nodes (*n* = 14; 13%), bone (*n* = 11; 10%), distant soft tissue (*n* = 9; 8%), distant lymph nodes (*n* = 4; 4%), brain (*n* = 2; 2%), adrenal (*n* = 1; 1%), and small intestine (*n* = 1; 1%). Compared with HPV-related *CYLD*-wildtype head and neck carcinoma, *CYLD*-mutant cases were sequenced much more frequently from liver samples (24% [35/148] vs. 7% [37/555], *p* < 0.0001; Table 1). Of the entire cohort of 148 *CYLD*-mutant carcinomas, known primary sites included oropharynx (*n* = 121; including 51 at the base of tongue, 51 tonsillar, 18 oropharynx not further specified, and 1 soft palate), nasopharynx (*n* = 3), and sinonasal tract (*n* = 1, which was an HPV16 positive case of sphenoid sinus origin). The remaining 23 cases were sequenced from metastatic samples, and the precise primary tumor site within the head and neck was not further specified.

**Table 1:** Comparative demographics, genomic alterations, and additional biomarkers of high-risk HPV-related head and neck carcinomas stratified by *CYLD* mutation status.

	*CYLD*-mutant	*CYLD*-wildtype	p*
Number of cases	148	555	
% male	94% (139/148)	87% (484/555)	0.02
Median age (range)	58 (38–89)	60 (8–89+)	0.684
Cylindroma-like histopathology (%)	**70% (98/141)**	**7% (34/459)**	**<0.0001**
Sequenced from liver metastasis (%)	**24% (35/148)**	**7% (37/555)**	**<0.0001**
Sequenced from lung metastasis (%)	20% (30/148)	15% (83/555)	0.131
Median TMB (Q1–Q3; mut/Mb)	2.61 (1.74–3.48)	4.35 (2.61–7.83)	**<0.0001**
HPV16 positive	95% (140/148)	89% (494/555)	0.043
MSI high	0% (0/138)	<1% (2/513)	1.000
PI3K/AKT/mTOR pathway			
*PIK3CA*	**11%**	**34%**	**<0.0001**
*KMT2D*^a^	**1%**	**16%**	**<0.0001**
*FBXW7*	**3%**	**11%**	**0.0018**
*PTEN*	12%	13%	0.679
*SOX2*^a^	2%	6%	0.085
*STK11*	2%	4%	0.442
Epigenetic regulation			
*KMT2C*	5%	11%	0.037
*EP300*	5%	6%	0.840
DNA damage			
*TP53*	4%	11%	0.008
*BCL2L1*	12%	5%	0.009
Single pass transmembrane receptor			
*NOTCH1*	6%	8%	0.598
Cell cycle regulation			
*RB1*	6%	7%	0.855
*CDKN2A*	1%	6%	0.019
*CCND1*	1%	3%	0.094
Receptor tyrosine kinase			
*FGFR3*	1%	7%	0.004
*ERBB2*	1%	3%	0.390
*EGFR*	0%	2%	0.081
Non-receptor tyrosine kinase			
*SRC*	5%	1%	0.015
RAS/MAPK pathway			
*KRAS*	1%	2%	0.320

^a^Limited data in the literature on role in PI3K/AKT signaling.

*The Bonferroni correction for 27 simultaneous comparisons was applied; rows with a significant *p* value (<0.0019) are highlighted in bold.

The *CYLD*-mutant carcinomas were clinically advanced. By AJCC 8th edition criteria [25], documented cases (*n* = 141) were predominantly stage IV (*n* = 135; 96%), while the remaining documented cases were stage II (*n* = 5) and III (*n* = 1). Stage was unknown for seven cases.

HPV typing by CGP then BLASTn revealed that *CYLD*-mutant and *CYLD*-wildtype HPV-positive carcinomas showed similar HPV16 predominance (95% [140/148] vs. 89% [494/555], respectively, not significant with corrected *p* value; Table 1). The remaining eight *CYLD*-mutant cases contained HPV18 (*n* = 2), HPV33 (*n* = 4), or HPV35 (*n* = 2) sequences. *CYLD*-wildtype cases showed comparable rates of HPV18 (5.0% [28/555], *p* = 0.064), HPV33 (2.5% [14/555], *p* = 1.0), and HPV35 (2.0% [11/555], *p* = 1.0) sequences detected.

In comparison with HPV-related *CYLD*-wildtype head and neck carcinoma, *CYLD*-mutant cases were sequenced more frequently from liver samples (24% [35/148] vs. 7% [37/555], *p* < 0.0001) (Table 1). Sample frequency from other sites, such as lung, was not statistically different between the *CYLD*-mutant and *CYLD*-wildtype cohorts (Table 1).

### Histopathologic features of *CYLD*-mutant hrHPV-positive HNSCC

Retrospective histopathologic review of 141 available *CYLD*-mutant cases, consisting of representative sections of 107 core or incisional biopsies and 34 excisional specimens, revealed SCCs with multiple patterns. Histopathologic patterns were grouped as follows: SCC with cylindroma-like features, nonkeratinizing SCC, predominantly nonkeratinizing SCC, and keratinizing SCC.

**Fig. 1:**
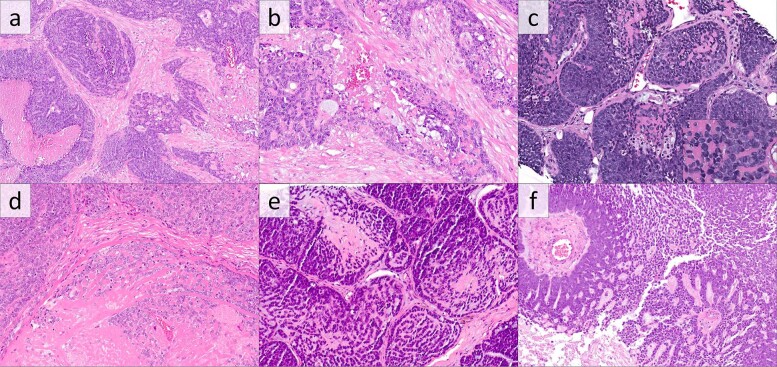
*CYLD*-mutant hrHPV-positive squamous cell carcinomas of the head and neck with cylindroma-like histopathologic features. **a**, **b** Carcinoma, nonkeratinizing, with crowded aggregates of tumors cells with comedonecrosis and round inclusions of basement membrane material. Higher power examination (**b**) reveals focal mucinous stromal material associated with the hyaline inclusions (H&E). (**c** and inset) Carcinoma composed of closely admixed and irregular tumor lobules with inclusions of basement membrane-like material and consisting of cells with scant cytoplasm and hyperchromatic nuclei (H&E). **d** Carcinoma with prominent basement membrane inclusions and necrosis with somewhat paler chromatin than other examples (H&E). **e** Carcinoma with closely apposed lobules with cribriform arrangement containing hyaline membrane and myxoid material (H&E). **f** Rare pattern of *CYLD*-mutant, HPV-positive carcinoma with cribriform pattern, mucinous stromal material, and limited inclusions of basement membrane material (H&E).

**Fig. 2:**
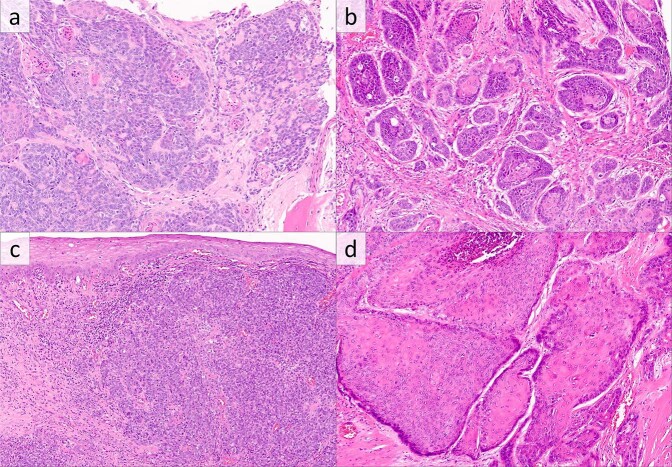
*CYLD*-mutant, hrHPV-positive squamous cell carcinomas of the head and neck with less common histologic patterns. **a** Carcinoma showing both cylindroma-like inclusions of basement membrane material and squamous morules and pearls (H&E). **b** Predominantly nonkeratinizing carcinoma with basaloid cytomorphology and limited foci of squamous differentiation (H&E). **c** Nonkeratinizing SCC (H&E). **d** Keratinizing squamous cell carcinoma with glassy eosinophilic cytoplasm (H&E).

The majority of *CYLD*-mutant SCCs (98 cases, 71%) displayed cylindroma-like histologic features, defined principally by the presence of inclusions of basement membrane material within aggregates of tumor cells. All cases, by definition for this subgroup, contained inclusions of densely hyalinized material, typically globular, consistent with basement membrane inclusions, which were occasionally in continuity with surrounding thickened basement membranes (Fig. 1). These basement membrane inclusions were widely distributed in 52 cases (53% of 98; Fig. 1a, b) and focal in 46 (47%) cases (Fig. 1c). Tumor necrosis was present in 88 cases (90%; Fig. 1a, d–f). All cases were composed of lobules of crowded tumor cells with generally “basaloid” cytomorphology, with cells containing scant cytoplasm and atypical nuclei. In total, 86 cases (88%) contained closely apposed tumor lobules. Foci of squamous differentiation, defined by keratin pearls or foci of aggregated squamous cells with dense eosinophilic cytoplasm, were present in 23 of these cases (23% of 98; Fig. 2a). Squamous differentiation was focal in 19 cases and multifocal in 4 cases. Myxoid stroma (stromal mucinous material) was present in 52 cases (53% of 98); in 36 cases, the myxoid material was directly admixed with the epithelial proliferations, while the myxoid material was limited to peritumoral fibrotic stroma in 16 cases. When associated with tumor cells, the myxoid material tended to be either interstitial or mixed with hyaline inclusions; only one case showed discrete inclusions of myxoid material (Fig. 1f).

For comparison, histologic features of 459 available cases of *CYLD*-wildtype, hrHPV-positive HNSCC were reviewed. Only 34 *CYLD*-wildtype HNSCC cases (7%) contained inclusions of basement membrane material. These inclusions were widely distributed in only three cases and focal in the other 31. In the context of this cohort of HPV-positive HNSCC, basement membrane inclusions were significantly associated with *CYLD* mutation (*p* < 0.0001; Table 1).

Of the 43 *CYLD*-mutant cases lacking basement membrane inclusions, histologic patterns were typical of HPV-positive oropharyngeal SCC [3], with patterns of nonkeratinizing SCC, predominantly nonkeratinizing SCC (nonkeratinizing SCC with focal maturation), and keratinizing SCC (Fig. 2b–d).

Twenty-two SCCs (16%) were nonkeratinizing, lacking apparent keratinization on the tissue examined. Of these nonkeratinizing SCCs, 11 cases (50%) showed necrosis, and deposition of myxoid material was present in 11 cases (50%), which was limited to stroma in 2 cases.

Ten SCCs (7%) were predominantly nonkeratinizing, with evidence of keratinization (squamous pearls or aggregates of cells with glassy eosinophilic cytoplasm) limited to small foci representing less than 10% of the examined tumor area. Seven of these cases (70%) showed necrosis, while six cases contained myxoid material, limited to stroma in one case.

Eleven SCCs (8%) were keratinizing with abundant squamous differentiation. Six cases showed necrosis, while three contained myxoid material, limited to stroma in two cases.

Review of immunohistochemical details in accompanying pathology reports found that the *CYLD*-mutant HNSCCs that had been evaluated by immunohistochemistry were consistently positive for p16 (99%; 76/77 cases), p63 (100%: 54/54 cases), p40 (100%; 39/39 cases), and CK5/6 (93%: 53/57 cases diffusely positive, 3/57 focal positive). CK7 was diffusely positive in only 2/21 *CYLD*-mutant cases, focally positive in 2/21 cases, and negative in the remaining 17 cases. *CYLD-*mutant HNSCCs were consistently negative when stained for synaptophysin (*n* = 0/35 cases), chromogranin (*n* = 0/33), TTF1 (*n* = 0/23), CK20 (*n* = 0/15), and CDX2 (*n* = 0/7).

### Comprehensive genomic profiling of *CYLD*-mutant hrHPV-positive HNSCC

**Fig. 3:**
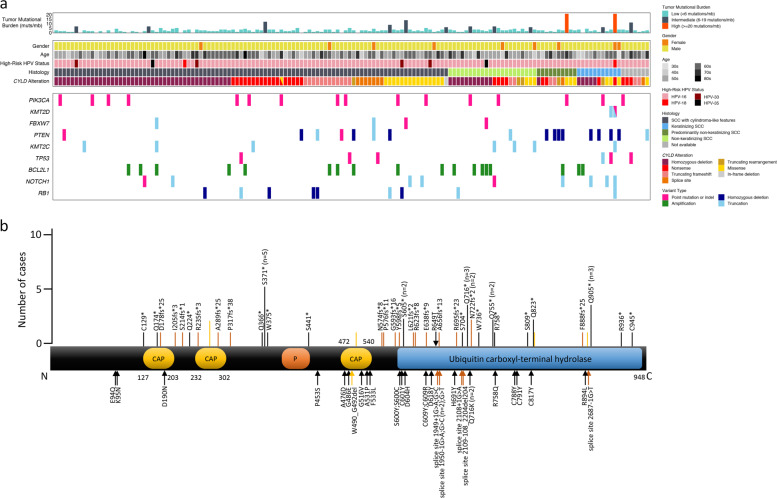
Clinicopathologic features and molecular landscape of HPV-positive *CYLD*-mutant head and neck squamous cell carcinoma. **a** Summary of clinical features, histopathology, and molecular alterations in *CYLD*-mutant head and neck carcinoma. **b** Schematic of functional domains of *CYLD* (transcript NM_015247), to include identified mutation sites. *CYLD* nonsense mutations (*n* = 29), frameshift mutations (*n* = 18), and truncating rearrangement site (*n* = 5) are denoted by black, orange, and yellow bars, respectively (top of diagram; length of bar~number of cases). Missense mutations (*n* = 25), splice-site mutations (*n* = 9), and in-frame deletions (*n* = 1) are labeled with black, orange, and yellow arrows, respectively (predominantly lower diagram). In total, 61 cases with homozygous deletions in *CYLD* are not shown. CAP cytoskeleton-associated proteins; P phosphorylation region.

The set of *CYLD* mutations in our cohort consisted of homozygous deletions (*n* = 61/148; 41%), nonsense mutations (*n* = 29; 20%), truncating frameshifts (*n* = 18; 12%), truncating rearrangements (*n* = 5; 3%; total truncating mutations = 52/148; 35%), missense mutations (*n* = 26; 18%), splice-site mutations (*n* = 9; 6%), and in-frame deletions (*n* = 1; 1%) (Fig. 3a, b). One *CYLD*-mutant tumor had a missense mutation and a nonsense mutation (Fig. 3a) and is counted in each group.

Truncating mutations occurred in exons 4 (*n* = 1), 5 (*n* = 8), 6 (*n* = 1), 8 (*n* = 1), 9 (*n* = 7), 10 (*n* = 2), 12 (*n* = 6), 13 (*n* = 3), 14 (*n* = 1), 15 (*n* = 1), 16 (*n* = 7), 17 (*n* = 3), 18 (*n* = 4), 19 (*n* = 2), and 20 (*n* = 5). Missense mutations occurred in exons 4 (*n* = 2), 5 (*n* = 1), 10 (*n* = 3), 11 (*n* = 3), 12 (*n* = 4), 13 (*n* = 4), 15 (*n* = 1), 16 (*n* = 2), 17 (*n* = 1), 18 (*n* = 3), and 19 (*n* = 1).

Figure 3a and Table 1 show the most frequent genomic alterations outside the *CYLD* locus in *CYLD*-mutant versus *CYLD-*wildtype HNSCC tumors. The *CYLD*-mutant group showed substantially lower TMB and less frequent alterations in *PIK3CA, KTM2D*, and *FBXW7*, all three of which are involved in the PI3K-AKT-mTOR pathway (Table 1). Alterations in *PTCH1* and *SMO* were rare or absent from the *CYLD*-mutant (1% and 0%, respectively) and *CYLD*-wildtype (1% and 0%) groups.

*CYLD-*mutant cases with predominantly nonkeratinizing SCC (SCC with focal maturation) (*n* = 10) and keratinizing SCC (*n* = 11) patterns showed significantly higher frequency of *PTEN* alterations compared with other *CYLD-*mutant cases, with four *PTEN*-mutant cases in each histopathologic pattern (38% [8/21] vs. 7% [8/120], *p* = 0.0004; dark green and blue versus dark gray and light green histology tiles in Fig. 3a). These cases also exhibited a higher frequency of alterations in the adjacent *FAS* gene (24% [5/21] vs. 3% [3/120], *p* = 0.0019). Demographics and site of sequenced specimen (primary vs. metastases) were similar between cases with each histologic pattern. We found no significant genetic differences between *CYLD*-mutant carcinomas with cylindroma-like features (*n* = 98) versus *CYLD*-mutant nonkeratinizing SCC (*n* = 22) (dark gray versus light green histology tiles in Fig. 3a). No *CYLD*-mutant or *CYLD*-wildtype cases demonstrated the *NFIB*-*MYB* gene fusion typical of adenoid cystic carcinoma.

Patients with *CYLD*-mutant tumors sequenced from primary (*n* = 41) vs. metastatic sites (*n* = 107) showed similar age and sex. Cases sequenced from metastatic sites showed a significantly elevated rate of *BCL2L1* amplification compared with primary-site tumor samples (16% [17/107] vs. 2% [1/41], *p* = 0.0248), particularly in cases sequenced from liver metastases (20%; 7/35 cases). Median TMB was not significantly different between primary-site versus metastatic tumor samples (1.7 vs. 2.6 mut/Mb, *p* = 0.072). No identifiable genetic differences were seen between specific primary anatomic origins of HNSCC (oropharynx, nasopharynx, sinonasal tract), but the number of non-oropharyngeal cases was limited.

Six *CYLD-*mutant cases underwent analysis for mutational signatures. Five of these cases were identified with an APOBEC signature. Of the available *CYLD*-wildtype cases, 208 underwent mutational signature analysis and showed a similar frequency of the APOBEC signature (63%, *n* = 131/208).

Comparison of cases with *CYLD* mutations within the ubiquitin hydrolase domain (exons 12–20) versus outside the protease domain (exons 4–11) revealed no significant clinical, histologic, or genomic differences. Within our *CYLD*-mutant cohort, cases with HPV16 sequences showed no significant differences when compared with cases carrying other hrHPV reads (HPV18, HPV33, or HPV35), although numbers in this latter group were limited.

Twenty of the 148 *CYLD*-mutant cases had available SGZ algorithm data to predict germline status, and all 20 *CYLD* mutations were predicted to be somatic.

Comparison of *CYLD*-wildtype HNSCC with cylindroma-like inclusions vs. those without these inclusions revealed that cases with cylindroma-like features showed significantly lower TMB (median 2.6 vs. 4.4 mut/Mb, *p* = 0.0219), and a lower frequency of *PIK3CA* (12% [4/34] vs. 35% [150/425], *p* = 0.0042), *KMT2D* (3% [1/34] vs. 18% [76/425], *p* = 0.0284), and *FBXW7* genomic alterations (0% [0/34] vs. 13% [53/425], *p* = 0.0229). In these parameters, *CYLD*-wildtype HNSCC with cylindroma-like histologic features resembled *CYLD*-mutant HNSCC, which often showed cylindroma-like features. In addition, *CYLD*-wildtype HNSCC with cylindroma-like histology showed a higher frequency of *NFKBIA* truncating alterations (12% [4/34] vs. 1% [4/425], *p* = 0.0014). No other significant differences between *CYLD*-wildtype HNSCC with vs. without cylindroma-like features were identified.

## Discussion

Here, we report that the presence of *CYLD* mutation defines a relatively frequent subset of HPV-related HNSCC that exhibits histopathologic features reminiscent of cylindroma and distinctive genomics. Our group recently demonstrated an analogous subset of HPV-positive, *CYLD*-mutant basaloid anal carcinomas with cylindroma-like histologic features [16]. In addition to similar histology, the HPV-positive *CYLD*-mutant anal carcinoma and HPV-positive *CYLD*-mutant head and neck carcinoma share unusually low TMB, low frequency of *PIK3CA* mutation, and a possible predilection for liver metastases.

Our current findings in HPV-positive HNSCCs correlate and may unify previously reported genetic and histopathologic findings for the *CYLD*-mutant subtype of HNSCC. A recent detailed genetic study using TCGA data found that one-third of HPV-positive SCCs of the head and neck contained mutually exclusive mutations of either *CYLD* or *TRAF3*, each of which is normally an endogenous inhibitor of NF-kB. Thus, these mutations were found to upregulate NF-kB activity [15]. HPV-positive SCCs with these mutations demonstrated several additional distinctive features, including HPV DNA in episomal form, rather than chromosomally integrated, and high levels of mRNAs associated with motility and proliferation. These *CYLD-* and *TRAF3*-mutant cases also showed improved clinical survival compared with HPV-positive HNSCC tumors lacking mutations in these genes. TMB was not reported. Status of the *PIK3CA* gene was examined in 36 HPV-positive HNSCC tumors (4 *CYLD*-mutant, 9 *TRAF3-*mutant, 23 *CYLD-*wildtype/*TRAF3*-wildtype), and *PIK3CA* mutations tended to be mutually exclusive from *CYLD/TRAF3* mutations. While this detailed study did not report histopathology, our series correlates HPV-positive, *CYLD*-mutant SCCs in this anatomic location with cylindroma-like histologic features, particularly the presence of basement membrane inclusions.

Earlier mRNA expression profiling and cluster analysis [26], as well as RNA sequencing studies [27], identified two distinct subtypes of HPV-positive HNSCC characterized by differential mRNA expression, copy number alterations, HPV DNA integration into host chromosomes, and frequency of *PIK3CA* mutations. One subtype (termed HPV-classical or HPV-keratinocyte, HPV-KRT) was characterized by expression of genes in keratinocyte differentiation, high levels of host chromosomal integration of HPV DNA, gain of chromosome 3q, frequent *PIK3CA* mutations, and keratinization on histopathology. The other subtype (termed HPV-immune response, HPV-IMU) was characterized by expression of genes implicated in immune responses and mesenchymal differentiation, low frequency of host chromosomal integration of HPV DNA, infrequent *PIK3CA* mutations, loss of chromosome 16q, and infrequent keratinization [26, 27]. Interestingly, *CYLD*, which was listed in the supplemental data of reference [27] along with a large number of other genes on chromosome 16q, showed somewhat decreased expression in the HPV-IMU group [27]. Correlating these prior studies [15, 26, 27] with our current findings, we note that our cohort of HPV-positive, *CYLD*-mutant HNSCCs shares some key features with the HPV-IMU subtype of HNSCC. In particular, we found that *PIK3CA* mutations are significantly less frequent in *CYLD*-mutant than in *CYLD*-wildtype HPV-positive HNSCC, and that the majority of these cases demonstrate histopathologic features of crowded tumor cell aggregates with basement membrane inclusions and limited keratinization (Fig. 1). In addition, as noted above, Hajek et al. showed that *CYLD*-mutant HNSCC generally lacked chromosomally integrated HPV DNA [15], another feature of the HPV-IMU subtype of HNSCC [26, 27].

From a morphologic standpoint, many cases in our cohort of HPV-positive, *CYLD*-mutant HNSCC display histopathologic features of the basaloid variant of HNSCC [4–6], suggesting that *CYLD* mutation may be a marker of HPV-positive basaloid variant SCC. Basaloid SCC is composed of crowded aggregates of cells with scant cytoplasm and with associated glassy eosinophilic material surrounding tumor lobules and entrapped as small round inclusions, variably termed “hyalinosis” or “hyaline stromal cores.” The jigsaw puzzle-like pattern of closely situated irregular lobules of tumor cells and the presence of hyaline material deposition have been emphasized as features that distinguish the basaloid variant of SCC from the more common nonkeratinizing HPV-associated HNSCC [6, 8, 28]. In a classic study of ten basaloid SCCs of the tongue, hypopharynx, and larynx, Wain et al. defined basaloid SCC as an admixture of basaloid and squamous foci: basaloid areas consisted of densely arranged small cells with scant cytoplasm showing lobular growth, small cystic spaces containing myxoid material, and collections of hyaline material, while squamous zones manifested as either surface SCC in situ or an invasive component with typical keratinizing features such as keratin pearls and prominent intercellular bridges [4]. Some cases displayed a cribriform pattern of epithelial cells with matrix material likened to adenoid cystic carcinoma. By electron microscopy, cystic spaces contained either loose granules or replicated basement membrane material. In a later study of 40 similar cases, Banks et al. identified the hyaline material surrounding and within tumor lobules in approximately two thirds of cases and further noted the frequent presence of comedonecrosis [5]. Comparative study of oropharyngeal and laryngeal/hypopharyngeal basaloid SCCs has shown similar histologic features, including deposits of hyaline material and necrosis [29]. To the authors’ knowledge, *CYLD* status has not been reported previously for histopathologically well-characterized subtypes of HNSCC [4–6, 29, 30].

The association of histopathologic features of the basaloid variant of SCC, particularly hyaline deposition, with *CYLD* mutation suggests a novel correlation between genotype and histologic phenotype in HNSCC. Our impression, especially in light of *CYLD* mutation, the fundamental alteration in familial and sporadic cylindromas [13, 14], is that the deposition of this hyalinized basement membrane material most resembles that seen in cylindroma. To the authors’ knowledge, study of genomic alterations within the basaloid variant of HNSCC is limited to a 2004 study of HNSCC subtypes, including seven basaloid carcinomas, using microsatellite markers which found that basaloid SCC tended to show loss of heterozygosity at loci on 9p and 11q [30]. Interestingly, *CYLD*-wildtype carcinomas with cylindroma-like basement membrane inclusions showed strikingly similar genomics to *CYLD*-mutant cases, with similarly low TMB and significantly less frequent alterations in *PIK3CA*, *KMT2D*, and *FBXW7*. Furthermore, these cases were enriched for inactivating alterations in *NF-kappa-B inhibitor alpha* (*NFKBIA*), leading us to consider that cylindroma-like features in HNSCC may be associated with non-*CYLD* mutations that activate related pathways. These potential mechanisms of histogenesis, including *TRAF3*, which was not sequenced in this study, require further investigation.

Studies of the prognosis of basaloid SCC of the head and neck have found variable results, ranging from worse to improved prognosis for the basaloid subtype [31–34]. These studies are complicated, however, by the variable presence of HPV infection, which is common in the oropharynx and which plays an important role in prognosis of HNSCC [6, 9]. Furthermore, some of these prognostic studies did not clearly delineate nonkeratinizing oropharyngeal SCC, sometimes called “basaloid” SCC in earlier studies, from the bona fide basaloid variant of SCC.

With respect to HPV infection among basaloid SCCs of the head and neck, Begum et al. found that 76% of basaloid SCCs occurring in the oropharynx were HPV-positive while nonoropharyngeal cases (hypopharynx, larynx, oral, and sinonasal) were rarely positive for HPV [9]. Chernock et al. similarly demonstrated consistent HPV positivity by in situ hybridization among basaloid SCCs occurring in the oropharynx, while laryngeal and hypopharyngeal cases were consistently negative [6]. Both studies found that HPV-positive cases have improved prognosis compared to HPV-negative cases [6, 9]. The finding by Hajek et al. [15] that HPV-positive, *CYLD*-mutant HNSCCs demonstrate a more favorable prognosis than *CYLD*-wild-type cases suggests that *CYLD* mutation, or potentially the surrogate of correlated histopathologic features, may inform the clinical phenotype of HPV-positive HNSCC.

CYLD, a de-ubiquitinating enzyme, suppresses both the NF-κB pathway [35, 36] and the c-Jun N-terminal kinase pathway [37–39]. Experimental models have shown that CYLD loss promotes aggressive behavior in various malignancies, including cutaneous SCC [40], pancreatic carcinoma [41], and hepatocellular carcinoma [39]. *CYLD* mutation has been most extensively studied in the context of cutaneous cylindromas in both syndromic manifestations, such as Brooke–Spiegler syndrome, and sporadic tumors [13]. A variety of mutations of *CYLD* have been characterized, typically resulting in truncation of the encoded enzyme [14, 42–44].

Morphologically similar lesions, such as adenoid cystic carcinoma or salivary basal cell adenocarcinoma, may arise in the oral cavity and oropharynx [45, 46], necessitating awareness of oropharyngeal SCC variants, including those with cylindroma-like features and *CYLD* mutation. The histopathologic presence of necrosis, mitotic activity, and nuclear pleomorphism favors basaloid variant SCC, and by analogy the cylindroma-like carcinoma described here, over adenoid cystic carcinoma [47].

Basal cell adenocarcinoma, which usually arises in the parotid gland but may develop in minor salivary glands, shares with the *CYLD*-mutant HNSCCs in this study several features including basaloid cellular composition, increased basement membrane material with inclusions, and, as suggested here, *CYLD* mutation. Both tumor types also display peripheral palisading of nuclei and foci of necrosis [48]. A recent study identified *CYLD* mutations within both basal cell adenomas and basal cell adenocarcinomas, perhaps accounting for some of the morphologic overlap with the cases in this current study [49]. Another study found *CTNNB1* mutations in basal cell adenomas and, among other alterations, focal *CYLD* deletion and biallelic inactivation of *NFKBIA* in a case of basal cell adenocarcinoma [50]. Although few cases of HPV testing of basal cell adenocarcinoma have been reported, rare tested cases have been HPV-negative [51], contrasting the HNSCC cases in this study. Furthermore, basaloid SCCs are generally negative for CK7 and CD117, aiding in the distinction of basal cell adenocarcinoma of the salivary gland [47].

By immunohistochemistry, basaloid variant SCC of the head and neck is p63 positive and usually positive for keratins, albeit sometimes focally [52]. Tumors are negative for synaptophysin and chromogranin, which can be useful for distinguishing basaloid SCC from small cell carcinoma [10, 52]. The positivity for p63 and negativity for neuroendocrine markers also distinguishes basaloid SCC from the rare entity of HPV-associated neuroendocrine carcinoma of the oropharynx, which has the opposite immunophenotype [53, 54]. Interestingly, a recent study of basaloid SCC showed that a majority of tumors were positive for SOX10 [55], a feature shared with most cylindromas [56, 57], as well as some many other cutaneous adnexal tumors and salivary gland tumors [58].

Cylindrocarcinomas of the skin are extremely rare and, ultimately, dissimilar to basaloid HNSCC. While both are carcinomas related to *CYLD* alterations, cylindrocarcinoma consists of a malignant proliferation associated with a precursor cylindroma [59]. Although *CYLD* mutations have been shown to be present in sporadic and syndromic cylindromas [13], molecular characterization of cylindrocarcinomas, beyond their occasional association with syndromes of germline *CYLD* mutation, is limited to characterizations of *TP53* alterations [60, 61].

In our assessment, basaloid carcinoma of the anus is closely analogous to the *CYLD*-mutant oropharyngeal carcinomas described in this study. In a 2005 report of basaloid carcinomas of the anus, Chetty et al. noted that the histologic features of basaloid cytomorphology, comedonecrosis, and basement membrane inclusions were shared by both the basaloid anal carcinomas and those occurring in the oropharynx [62]. In addition to these overlapping histopathologic changes, we further note that these carcinomas from both sites show consistent *CYLD* mutations, HPV positivity, low TMB, low frequency of *PIK3CA* mutations, and likely propensity to metastasize to the liver [16].

Limitations of our study primarily concern the nature of our cases, which were drawn from a database of aggressive and advanced stage (AJCC 8th edition) cancers sent for analysis to search for potentially targetable genetic alterations. Thus, indolent tumors or those cured by excision were highly unlikely to be included. Follow-up data were also limited, and thus future studies will be needed assess the clinical course of these cancers, including prognostic features and therapeutic responses. *TRAF3* mutation and HPV genomic integration were not evaluated in this study. In addition, we were able to review only the histopathology of the sequenced tissue, representing one block of tumor, most often that of a core or incisional biopsy, potentially limiting the ability to detect focal histologic features. Because the initial histologic review focused on the *CYLD*-mutant tumors to establish the range of histologic features, thereby prompting a later comparative review of *CYLD*-wildtype cases, *CYLD* mutational status was known at the time of review, presenting the possibility of observer bias. Finally, immunohistochemistry was not available for evaluation beyond review of details from outside pathology reports.

In summary, we found that over one in five HPV-positive head and neck carcinomas in our cohort carry *CYLD* mutations, and that the presence of *CYLD* mutations defines a subset of HNSCC tumors that often display cylindroma-like histopathologic features and exhibit unusually low TMB and infrequent alterations in *PIK3CA*. Characterization of HPV-positive, *CYLD*-mutant carcinoma may assist in classification of HNSCC and potentially inform prognosis and experimental therapies for the patients affected [17, 18], both topics that will require additional study.

## Supplementary information

Supplemental Figure 1

Supplemental Table 1
