# Supplementary material for: CYLD mutation characterizes a subset of HPV-positive head and neck squamous cell carcinomas with distinctive genomics and frequent cylindroma-like histologic features
Source: Mod Pathol. 2020 Sep 5;34(2):358–70. doi: 10.1038/s41379-020-00672-y (PMC7817524; doi:10.1038/s41379-020-00672-y)
Supplement: Supplementary file 1 — Supplemental Figure 1 [file 41379_2020_672_MOESM1_ESM.pdf]

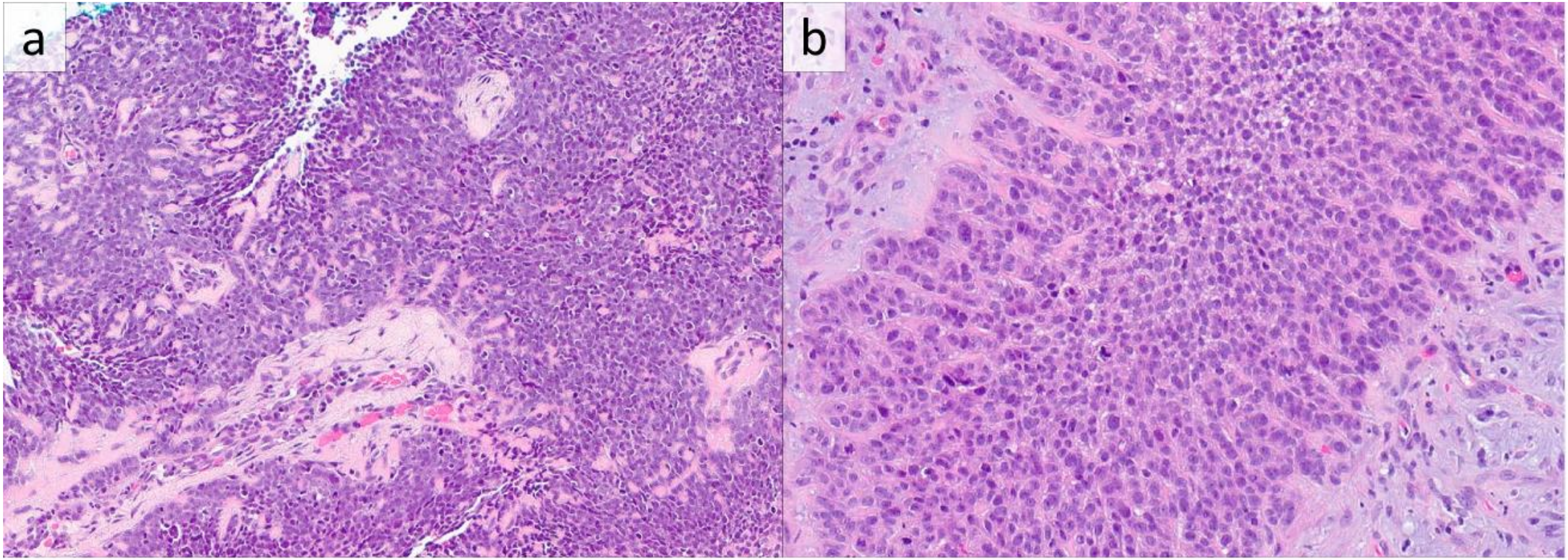

Supplemental Figure 1: *CYLD*-wildtype, HPV-positive HNSCC with cylindroma-like inclusions. **a**) Carcinoma with basaloid cytomorphology and frequent inclusions of basement membrane material (H&E). **b**) Carcinoma with basement membrane inclusions and associated stromal mucin (H&E).
